# Life Events and Adaptive Coping Approaches to Self-Management From the Perspectives of Hospitalized Cardiovascular Patients: A Qualitative Study

**DOI:** 10.3389/fpsyt.2021.692485

**Published:** 2021-09-16

**Authors:** Ruolin Qiu, Leiwen Tang, Xiyi Wang, Zhihong Ye

**Affiliations:** ^1^Affiliated Sir Run Run Shaw Hospital, Zhejiang University School of Medicine, Hangzhou, China; ^2^School of Nursing, Shanghai Jiao Tong University, Shanghai, China

**Keywords:** cardiovascular diseases, life events, adaptation, self-management, qualitative research

## Abstract

**Objectives:** This study aimed to explore the association between hospitalized cardiovascular patients' life events and adaptive coping approaches to self-management.

**Methods:** The study was a qualitative study that was conducted in a cardiology department of one affiliated university hospital in Hangzhou, China. Twenty-eight participants with cardiovascular diseases were recruited through a purposive sampling procedure. Semi-structured interviews were used to gain insights into adaptive coping approaches to self-management when living with different life events. Interviews were audio-recorded and transcribed, and the data were analyzed by thematic analysis.

**Results:** Life events reported by hospitalized cardiovascular participants could be summarized in four categories: daily routines, life changes, life-threatening experiences, and emotional sufferings. The adaptive coping approaches were also summarized in four themes: decision-making, avoidance, consistent responses, and episodic responses.

**Conclusion:** This study described essential insights into the mutual influences between various life events and adaptive coping approaches to self-management by a group of hospitalized cardiovascular patients. Participants coped with their problems flexibly by processing comprehensive information from various and unpredictable life events regarding the situations and contexts. While inequity was cumulated, psychological resilience was a vital mediator between stressful events and their responses. The study illuminated the importance of understanding context, situations, and experiences on how cardiovascular patients adapted to their self-management regimens.

## Introduction

Among chronic diseases, cardiovascular disease is one of the leading causes of mortality and results in different kinds of burdens for the government, family, caregivers, and individuals (1–4). Self-management is described as an interactive and everyday process, involving environmental, physical, mental, and other domains, which is widely recognized as a convenient and economical method to monitor chronic diseases and maintain a satisfactory quality of life (5–7). However, despite effectiveness of self-management, it is dynamic, depending on the stages or phases of cardiovascular disease and other kinds of changes in the patient's life situations and contexts (8). Thus, to improve the quality of life, cardiovascular disease patients should accordingly adjust their self-management strategies over time across their life span.

Life events occur in a real-world setting and accumulate during the life span, which has a great impact on individuals' well-being, health, and behaviors; and evidence in the literature has shown that stressful life events are highly correlated with disease and health outcomes, which could be defined by epidemiological, psychological, and biological traditions (9). Therefore, coping with different life event situations is an essential part of long-term life adaptation to kinds of human diseases (10–12). Besides, resilience represents coping and rising above difficult experiences, indicating the capacity of a person to successfully adapt to change after stressful situations and resume the previous so-called healthy conditions psychically and mentally (13). Self-management is a complex set of various life events and includes not only the normative aspects but also the stressful challenges. Studies have previously investigated the relationship between stressful life events and self-management within some populations with chronic diseases (e.g., diabetes, metabolism, and chronic obstructive pulmonary disease), like groups of children and old rural patients (14–16). As for cardiovascular disease, quantitative studies have examined the coping strategies (17), as well as their associations with anxiety and physical functioning in heart failure groups (17, 18). And there were also qualitative studies exploring life experiences and coping strategies in patients with heart diseases (19–21). A longitudinal qualitative study has developed four chronic illness self-management patterns over time from a broad perspective (8) but did not specifically introduce the relationship between life events and self-management. Researchers have found that the use of maladaptive coping strategies can divert attention from heart failure among older patients (22). A literature review taken by Li and Shun (23) has discussed the coping styles of patients with chronic heart failure, like emotion-focused and problem-focused coping, but it did not explore all types of cardiovascular patients. Moreover, another qualitative meta-synthesis integrated information supporting coping and adaptation of left ventricular assist device (LVAD) patients, which had four stages including different tasks in physical, psychological, and social domains (24).

Although there were several studies that have explored the relationship between coping and life events, there is little known about the coping strategies for living with cardiovascular diseases in samples of Chinese hospitalized patients. Thus, capturing these aspects from the participants' viewpoints is necessary for tailoring interventions that could promote better self-management adaptation effectively in cardiovascular groups. Hence, this study aimed to explore the patients' life events and adaptive coping approaches to self-management when living with cardiovascular diseases.

## Methods

### Design

A qualitative design was applied, which aimed to explore cardiovascular patients' life events and their adaptive coping approaches for self-management. Semi-structured interviews were chosen to collect rich data. Thematic analysis (25) was used to extract the themes and develop results to learn more about patients' adaptive coping strategies for self-management when living with their chronic cardiovascular conditions.

### Setting and Sampling

The study was undertaken in a cardiology department of one affiliated general university hospital with 2,400 beds in Hangzhou, China. After the authors obtained permission to enter the department from the Nurse Deputy Director and Department Nursing Directors, study recruitment began.

A purposive and criterion-based sampling procedure was used to recruit patients with rich information. The criteria selecting the participants were included as follows: (1) aged 18 years or older; (2) living with chronic diseases over 6 months and having at least one cardiac event; (3) willing to share their experiences and to express themselves clearly; (4) volunteered to provide informed consent; and (5) has no involvement in other studies at the same time. Nurses in the recruitment site assisted in identifying eligibility, including registration information and medical history. Potential participants' names and bed numbers were collected, and then nurses informed them about the study. The specific time that authors contacted potential informants depended on the availability of patients and/or their families, usually after daily ward rounds. Considering the time constraints of staying in the department, when researchers found no more new valuable information and data reached information saturation, they had a discussion to achieve a consensus on the time point of stopping recruitment and leaving the field. Thus, a total of 32 patients under hospitalization were invited, and 29 persons agreed to participate in the study. One female patient refused the invitation because she wanted her disease to be kept a secret. Six invitations were sent to participants' family members, and two of them rejected the invitation directly. Moreover, one male patient finished the interview but was still excluded owing to the complicated dialect. As a result, data comprising 28 participants were finally analyzed.

### Data Collection Procedure

Selected individuals received an information letter inviting them to participate in the study and an oral introduction of the study before the interviews was carried out, including the purpose, guarantee of confidentiality, and voluntary principle. They were invited to have face-to-face, in-depth interviews to share personal experiences once they were available. Under the study setting, participants chose a location in the department for their interviews according to individual preference, such as a quiet meeting room or a private corner. Some participants liked to stay in bed with curtains pulled up while receiving the interviews after interventional operations when no ward mates were present in the shared room, where it could be regarded as a relatively private place. Fourteen caregivers accompanied their patients in case of any emergent care needs. Besides, caregivers were only supposed to explain some local dialect expressions for the researchers when needed; otherwise, they were not permitted to influence the interview process.

Data were collected from April to May 2018 by two competent nursing students: one PhD student (RQ) and one master student (QZ). Both of them have received professional training for the techniques about how to conduct qualitative studies. Most interviews were mainly guided by RQ, with QZ as an assistant taking note, except for three interviews conducted by QZ. Based on the Chronic Care Model (26), data collections followed a semi-structured interview guide, which was piloted in patients with chronic diseases at the physical examination center and locally adjusted to have more understandable questions. Before the interviews, all participants were invited to provide informed consent. It was critical to emphasize the aim to learn different perspectives from patients about the research. Interviews started with warm greetings and self-introduction to develop a rapport before proceeding to more sensitive questions. Then questions like “Could you please share your experiences about how you live with cardiac diseases?” or “How do you cope with your body conditions?” aimed to allow participants adequate time to explore their experiences. Several probing questions, such as “Could you please tell me more about that thing you mentioned just now?” and “What did you exactly do when you encountered a severe heart attack,” were intentionally asked to encourage participants to clarify descriptions or provide rich information when narratives referred to different contexts and specific events. The questioning was relatively flexible rather than inducing any answers. Participants could use accents and their expressions to generate their stories. The final question was always a form of “Do you think there is anything else I should have asked or do you have anything else to add?” Most interviews took place at the bedside, between 5 and 115 minutes. Two patients (P15 and P19) were informed by the nurse to do examinations and were no longer available for the interview, and one of the interviews (P19) lasted only 5 minutes. However, the two participants, and the 5-min interview, provided rich information so that after consulting the expert and holding a discussion within the team-member group, the two narrative data were kept with the patients' permissions. Interviews were audio-taped and all transcribed verbatim in Chinese. Every participant was assigned a code to guarantee anonymity.

### Data Analysis

A thematic analysis was applied in the study according to the method approach recommended by Braun and Clarke (25). Qualitative data could be analyzed by hand or computer software; therefore, RQ, the first author, analyzed the data manually. Owing to the preference for analyzing by hand and a small number of the transcript pages, RQ used color markers to distinguish coding parts of texts. At the beginning of the analysis, the transcripts were read repeatedly for a better understanding, while some basic ideas related to life events and adaptive coping approaches were written down to obtain an overall picture. Then, qualitative data were reviewed in more detail, and text segments were bracketed and narrowed into codes, with the meaningful descriptions. Third, similar codes were separately collated into groups, and the major ones were retained by examining whether they were discussed most frequently by participants, most supportive, or most relevant to our topics. Without a preexisting frame, potential themes or subthemes focusing on life events and coping approaches were developed until no more new evidence provided additional themes. These major themes brought authors' perspectives to make interpretations of collecting qualitative information. Rest codes and candidate themes were made for further decisions to reach an accurate representation. Otherwise, redundancies were eliminated. Finally, clear definitions and detailed descriptions about the identified life events and sorted adaptive coping approaches for self-management were established to fit determined themes (25). During the analysis process, many memos were used for extracting and tracking the initial ideas.

### Rigor

To enhance the rigor, there were several strategies. First, having two researchers always present in the interviews, one mainly guiding the interview while the other taking notes, allowed different perspectives and conclusions. Then, opinions from cardiovascular participants with complications or multi-morbidities helped enrich the data source. Third, caregivers playing the roles of dialect interpreters improved the understanding of patients' descriptions. These strategies boosted the triangulation in this qualitative study. Besides, identified themes were discussed with an expert (KS) who had significant cultural differences and reviewed by a third team member (LW) for opinions. Peer review ensured credibility during the process of integrating the data. Furthermore, participants who wanted to know the findings received the transcribed documents and the initial results. In case of discrepancies, member checking was also a good method to solve these problems, and their opinions were considered in the final presentation to help confirm if the authors had specified the themes adequately and accurately.

## Results

### Patients

There were 21 men and seven women contained in this study, between 32 and 86 years old. All of them were admitted to the hospital due to a cardiac event. Everyone has been diagnosed with hypertension, and some of them also had diabetes, gout, and other chronic conditions. Most of the participants had received an education, and half of them went to college. Most participants were from Zhejiang Province, and some of them were retired. Detailed demographic characteristics are shown in Tables 1, 2.

**Table 1:** Patients' demographic characteristics.

**Variables**	**Total** (***N*** **= 28)**	**%**
**Age**		
Mean age	62.50	–
Range	32–86	–
**Gender**		
Male	21	75.0
Female	7	25.0
**Marital status**		
Married	24	85.7
Divorced	1	3.6
Widowed	3	10.7
**Education**		
No formal education	1	3.6
Primary	4	14.3
Secondary	11	39.3
College or university	12	42.8
**District**		
Zhejiang province	24	85.7
Other provinces	4	14.3
**Occupation**		
Private	6	21.4
Employed	10	35.7
Retired	12	42.9
**Religion**		
Christian	4	14.3
Buddhist	2	7.1
Atheist	10	35.7
Not reported	12	42.9
**Chronic conditions**		
Single cardiovascular disease	8	28.6
Multiple chronic conditions	20	71.4

**Table 2:** Details about patients' chronic conditions.

**Patient**	**Gender**	**Age**	**Chronic condition**
1	M	57	D, HL, HT
2	F	56	D, HT
3	F	61	B, CKD, HT
4	M	51	CVD, HT
5	M	52	HT(P)
6	M	79	HT
7	M	62	HT
8	M	34	HT
9	M	48	HT
10	F	70	CVD, HT
11	F	84	CVD, HT
12	M	72	HT, D, CVD
13	M	86	HT, G, CA
14	M	48	HT
15	M	73	HT, D, HL, CVD
16	M	52	HL, HT
17	M	40	D, HT, S
18	F	86	CVD, D, HT
19	M	32	HL, HT
20	F	45	D, HL, HT, S
21	F	79	CVD(P), D, HT
22	M	72	HT(P)
23	M	68	CVD, G, HT
24	M	69	CVD, HT, S
25	M	79	CVD, D, HT, S
26	M	77	HT
27	M	53	CVD, D, HL, HT
28	M	65	CVD, HT

*F, female; M, male; B, Behcet syndrome; CA, cancer; CKD, chronic kidney disease; CVD, cardiovascular disease; D, diabetes; G, gout; HL, hyperlipidemia; HT, hypertension; P, pacemaker; S, stroke*.

### Life Events

When describing the experiences of living with cardiovascular disease, patients described various life events, which could have occurred before, during, and after the disease diagnosis. Participants thought these events had a positive or negative impact on their self-management concepts and awareness or behaviors.

#### Daily Routines

Daily routines were the most cited statements in participants' narratives. As an almost fixed and frequently happening itinerary on participants' schedules, these kinds of reported events cover the fundamental aspects of daily life and were likely to have exact time points.

##### Normal Life Activities

Normal life activities referred to the most straightforward behaviors that were tightly related to cardiovascular participants' basic survival, such as eating, entertainment, and working. Several patients mentioned that they would like to have access to medical information through advanced techniques. The younger ones were interested in using mobile devices, while the older ones preferred traditional ways. P28 regarded watching a health-related TV program as a regular activity after work to learn more medical knowledge: “As soon as I get home, I open the TV to watch the ‘Meet on the road of Health' every day” (P28, male, 65). In addition, P10 reported that she likes watching health channels. Moreover, reading health magazines was her favorite daily work:

“We subscribed to a series of medical reading materials. […] Once we get free time, we will read these magazines. I usually gather those useful information parts, and make them a small scrapbook.” (P10, female, 70)

At the same time, even if some participants were over 70 years old, they have received a good education in their youth. Therefore, they could still follow the trend. Well-educated P12 was always enthusiastic about searching on the internet or watching online videos for specialist opinions, and P13 enjoyed connecting with his families and watching videos by pads.

“I searched for some information about the techniques of coronary arteriography on the internet, and I found two domestic websites, which speak more authoritatively nationwide.” (P12, male, 72)

As for eating, those who had more social activities tended to lead a more unhealthy lifestyle. Given that P9 was a driving coach, he used to be invited to have big meals in restaurants after his students obtained licenses. Therefore, in his late forties, he still kept the eating habit, for he thought he was only diagnosed with hypertension rather than other serious diseases: “I enjoy eating those kinds of oily stir-fried dishes, delicious and tasty” (P9, male, 48). Although the youngest informant clearly understood that he was leading an unhealthy life due to his job shift, he was unable to change this situation:

“If I am not available [for lunch] during shifts, I will just grab a bite to eat, like fried pancakes or other instant food.” (P19, male, 32)

##### Self-Care

Self-care is linked to enhancing a better lifestyle and quality of life. Some of these cardiovascular patients in current study considered that self-care was nested in their daily routines, and was one of the most frequent everyday events. Such events are always combined with normal life activities so that they could have better interactions with the context. Many participants reported that they were used to walking for a while in the early morning or after dinner. For example, a male participant with both diabetes and cardiac diseases told that it was a habit to do so:

“Sometimes in the morning, well, I get up at 5:00 or 5:30 am. [Take a deep breath] First, have the insulin injected- I should have the meal in half an hour –and then, walk for one and a half hours. Yep, walk, and then eat.” (P27, male, 53)

As for receiving acute care during hospitalization, participants said professional health providers and equipment made them rest. Meantime, they had realized the severity of these non-communicable diseases. Thus, it was rather natural for patients to have better performance, especially to follow the regular time of medication and meals, as well as quitting smoking and alcohol consumption. As P23 confirmed surely:

“After being admitted in the hospital, emm, I absolutely gave up smoking and did not pick it up anymore. And the drinking was also cut out for a long period.” (P23, male, 68)

#### Life Changes

Participants mentioned that they had encountered several significant life changes when living with their diseases, such as starting working, getting married, a family member passing away, and moving. As a successful and confident self-employed person, P1 stated that he always worked here and there, and once he worked and settled in one place, he gradually adapted having a local lighter flavored cuisine than in his hometown: “It was after I came to Hangzhou that I ate less oily food” (P1, male, 57). Except for this, P1 did not keep other dietary habits in check, such as alcohol consumption, and he said he seldom exercised. Although he was worried and aware of the importance of taking care of his body, he was too confident to follow the doctor's suggestions. P8 reported that his life had changed significantly than he expected, like divorcing his wife, his mother being seriously ill, and being a patient. Though life was so harsh, he was still in strong morale to face the difficulties from each aspect:

“I mean, my health condition's poor and I can't afford any heavy work, which makes me under huge pressure. […] Health matters to me. Ah! Yea… My wife and I have been divorced, and my child also left me. These are definitely stressful. […] So what? No matter how big the pressure is, you have to live.” (P8, male, 34)

Some uncontrollable events had significant impacts on society, leading to critical political or economic changes. These changes also brought differences to individuals' life, such as social status, financial conditions, and personal values, especially for those participants older than other informants. An old lady narrated a story about how she got hypertension in a political protest during the Cultural Revolution and how she dealt with the problem after that:

“Due to the human resource shortage, I was assigned to work for the Municipal Government such that I even couldn't take a good rest at that time. […] Though the blood pressure was still at a high level, I insisted on working rather than asking for sick leave.” (P18, female, 86)

#### Life-Threatening Experiences

Life-threatening experiences were reported to be a crucial time point for patients to realize that health management was related to cardiogenic shock, severe complications, and unexpected side effects. Under this situation, those participants who had unreasonable self-management always expressed their wish to manage themselves better, mainly middle-aged male participants. A participant described his experience as follows:

“I got a shock within 10 minutes after arriving at the outpatient department by myself, and then I was admitted to the hospital. I was in an unconscious status from February 19th till March 6th. Finally, I woke up, and it was just like a dream.” (P4, male, 51)

Any of these emergency situations would occasionally cause severe accidents and threaten participants' lives. Several patients said they had even been on the edge of death. P26 described that he had fallen from the upstairs, due to the dizziness caused by his hypertension: “I felt dizzy at first, and then I rolled down [from the third floor], from upstairs to downstairs” (P26, male, 77). Another event was told by P27: “Last year, I had a car accident, um… it might be a heart attack, yea, caused by a heart attack” (P27, male, 53).

#### Emotional Sufferings

Almost every participant admitted that they were always involved in emotional fluctuations, especially accompanied by symptom aggravations. Notably, hearing of others' sufferings made them realize that they were in the same position. Hence, they were led by emotions and would make decisions unhesitatingly. A story about how other patients struggled to have high-quality living but had poor outcomes would make them worry and would make their emotions churn. P26 learned lessons from relatives' death caused by a stroke, and shortly afterward, he decided to have a coronary stent to get rid of the horrific result. He acknowledged:

“My wife's brother had a stroke [and passed away]. […] What if I got a stroke? I am afraid of dying. […] Now, I am so pleased to have the interventional therapy.” (P26, male, 77)

Moreover, participants reported they were swept into the vortex of their embittered emotions while going through negative events like denial or rudeness from their attending doctors. In contrast, they said that they would like to behave better after receiving adequate support from professionals, peers, and families. Only seven women were recruited in this study, yet they told more emotional experiences than men. For instance:

“A doctor might think he humorously informed me, “Well, the disease is life-threatening, and you are extremely in danger!” I was frightened by what he told me. […] The information was true, but it was impolite to tell like that.” (P21, female, 79)

### Adaptive Coping Approaches to Self-Management

Most patients had to make adjustments related to their life due to the cardiovascular disorder. Based on different situations and event stimuli, potential benefits, and personal preferences, participants would develop optimal approaches to adapt to the current environment and situations. It was a status that patients chose a satisfying choice rather than the best one for themselves. These adaptive coping approaches could be either temporary, long-lasting, continuous, or intermittent.

#### Decision-Making

Cardiovascular patients admitted that they would gather kinds of information from their previous experiences, practice accordingly, and evaluate effectiveness. Participants with comorbidities tended to prioritize dominant self-management strategies to deal with the severest condition (e.g., myocardial infarction, and stroke), which meant that they could control the most obvious or threatening symptoms rapidly in an effective way; meanwhile, they might put less threatening changes aside (e.g., hypertension and diabetes). These processes generally occurred immediately after participants identified the severity and perceived the importance of adjustment, especially for those who had experienced terrible events. Take P12 as a decision-maker example: he used to bicycle to work and home, but he stopped riding after retirement and was living easy with his grandson. However, later he restarted and gradually strengthened exercising resulting from his glucose level, and at last, he was extremely careful about exercise intensity due to a terrible heart attack he had experienced. From then on, he mainly immersed himself into cardiovascular management:

“[…] I had high fasting plasma glucose which was 7 at first and my wife told me it was not that good. But I didn't care at all. One year later, the value increased to 8 and then up to 9 in the third year. At that time, I was finally in a panic and began to consult for information. […] I acknowledged, uh - they also told me as well- Uh, your diabetes would get better if you strengthened exercising. Later, that the same year, I had sudden angina. After that, I immediately transferred all my attention from diabetes to heart disease. […] Not joking, it was deadly once it was ignored.” (P12, male, 72)

Though participants would balance the risks and benefits to make an optimal decision, not all the decisions would prompt action. Several younger male participants even deeply understood the importance of a well-adjusted lifestyle and could have made a better change; nevertheless, they still chose to maintain an unhealthy status to make a living. They did not want to be a burden to the whole family; nor did they want to spend money given by their families. P17 was only 40 years old, but he had heart disease, diabetes, stroke, and other complications, any of which would remarkably influence his normal life, let alone working. Hence, he decided to prioritize his body with his wife's understanding, but he felt ashamed at the same time of being unable to make a living and had to rely on his wife:

“When I talked about this, I felt ashamed. (Forced smile) […] I don't think of anything else but staying here and improving my health condition. But my wife has to go to work, and there are heavy financial burdens.” (P17, male, 40)

P5 had recently undergone pacemaker insertion, and everything had changed fast. Even if his wife expressed her concerns, P5 still insisted he was in his prime: “I am considered to be the mainstay and breadwinner of the whole family” (P5, male, 52). After evaluating their conditions, some of the participants would make self-reflections, and then they would decide and plan for the adjusting frequency or degree of following responses. P4 was almost of the same age as P5 and had more awful experiences than P17—he was admitted into the ICU due to his severe disease conditions—but he did not receive any pressure from the medical expenditures, and as a result, he anticipated to focus more on his health rather than be a headstrong patient:

“After this unforgettable hospitalization, I must be more compliant with the doctor's suggestions and listen to my wife's advice about eating healthier at home.” (P4, male, 51)

In consideration of the long-term self-care condition of the cardiovascular disease, some compliant participants followed physicians' recommendations and recorded their daily results of blood pressure carefully in case there came any unpredictable situations. Thus, patients could have abundant and visible data for considerable decision-making.

“Sometimes the blood pressure level would drop a lot. Or if you noticed that the medicines didn't work and the value stayed at a high level, and then it was a great time point to ask the physician to change the prescription.” (P28, male, 65)

#### Avoidance

Some participants stated that they avoided practicing self-management regimens knowingly, such as smoking cessation and losing weight, which was recommended by many physicians for cardiovascular patients. They believed these behaviors would not cause any severe symptoms. Moreover, considering their personal preferences, they said they would like to satisfy themselves and would like everything to remain the same rather than perform those behaviors to restrict happiness. For instance:

“The doctors suggested that I should quit smoking, but it's hard to agree. Once I quit smoking, my life would become meaningless. Besides, the harm of smoking may not be as serious as the doctor said.” (P9, male, 48)

In contrast, patients who intended to modify their self-management strategies lacked confidence and were fearful of making a move forward. They described that their desires to make changes were sometimes hindered by those cumulative negative experiences. They reported they had encountered frustrating life events (e.g., unemployment and divorce) or annoying emotional feelings (e.g., denial and sadness) repeatedly during their self-management process. Female informants focused more on emotional description, whereas male participants concentrated more on factual statements. What is more, other uncontrollable factors (e.g., personality and policies) also impacted participants' minds. As a result, it was difficult for them to adapt to the current situation. For example, P17 was leading a smooth life before he lost his job. Soon after unemployment, he was diagnosed with multiple chronic diseases. The participant and his family endeavored to look for a better way to treat his diseases. While managing various conditions, life became tougher, and he lost his temper gradually. Aside from those incidents, he also suffered from several unhappy events when communicating with physicians. Cumulative disappointment and stubborn personality aside from his real economic situation all drove him to reject regular treatment but insist on undergoing folk therapy by himself:

“We have already got heavy financial burdens. […] The medical treatment in a top-class hospital would cost a lot, and we didn't have such a massive amount of money to cover the costs. There, it couldn't be better if these folk remedies cured my disease.” (P17, male, 40)

#### Consistent Responses

The patients had insisted on one behavior at a uniform time for years after the diagnosis of cardiovascular diseases without major adjustment, regardless of whether these behaviors were motivated passively or actively, such as regular exercise, periodic physical examination, and timed doctor visit. Patients' consistently compliance with the prescribed medication was the most frequently mentioned response to the treatment. They would willingly follow their doctor's suggestions and comply with the adjustment of the treatment regimen, and this behavior became a natural daily routine. When the disease conditions were under control and self-management benefits were accumulated, participants would strengthen their behavior over time. P16 described: “The doctor suggested that I should take antihypertensive drugs. It has been ten years since I started taking medication” (P16, male, 52). Other common consistent strategies for self-management were measuring blood pressure, eating healthy, and exercising. P22 mentioned his experience of using an electronic sphygmomanometer:

“Later, my son bought me an electronic one [sphygmomanometer], which is rechargeable and much handier, right? Once press the button, well, the screen will show the heart rate and it can tell you whether the result is in a normal range or not. Everything's displayed quickly and clearly, right? But I always forgot to use it, since I was not familiar with it at first. Eh, and then… I was skilled at measuring blood pressure so that it became my daily habit.” (P22, male, 72)

Cardiovascular patients who had multiple chronic used unique self-management regimens to manage their conditions. For P13, as a patient with cardiovascular disease and gastric cancer, his diet should be defatted after the gastrectomy: “I only eat that defatted food at home. […] My family buy coconut oil especially [for me], just because it can be absorbed by the intestines” (P13, male, 86). For those who had diabetes, blood sugar monitoring and insulin injection also were the main behaviors to perform: “Basically, I use the glucometer to test the blood sugar every day. In general, well… As soon as I get up, I will measure the blood sugar level, which is not over 8; and the level is also under control two hours after a meal, which will not exceed 14” (P26, male, 77).

#### Episodic Responses

The episodic responses referred to those self-management strategies that patients practiced occasionally, temporarily, or irregularly. Participants reported that they implemented different strategies depending on the surroundings and situations, like behaving distinctively in the medical institution and outside the medical institution. Hospitalization implied that they got acute symptoms and intense side effects; therefore, participants always attached great importance to the various suggestions given by professionals. Patients should have behaved in a good manner for a limited period. Nevertheless, P14 reported he was sincerely thankful for the treatment; however, he still argued with the physician due to a terrible outcome after a half month's hospitalization:

“I demanded a discharge in half a month because I was not treated in the right way, which contributed to my worse condition. […] I tried my best to keep a good mood to get rid of the bad effects.” (P14, male, 73)

P9 stated that he would follow the advised routines given by professionals while he was not so acquiescent at home. Nevertheless, he admitted he had just secretly eaten in a nearby restaurant: “I have just snuck out for lunch. […] The meal in the hospital seemed to be oil-free and I didn't have any appetite” (P9, male, 48). For another, P2 acted like a child to attract more attention from nurses, which she never did before: “It's kind of like a submissive kid who would like to follow the nurses” (P2, female, 56). Besides, some patients reported that they would perform well when accompanied by their family, especially those older people who lived by themselves, whether in the hospital or at home.

Additionally, participants were telling contradicting behaviors between home and other places except for medical institutions. P27 was fond of smoking and used to smoke within social contexts, whereas he described his own rule about respective behaviors outside and at home—never smoking in front of his families at home—which became a habit unconsciously:

“I set myself a rule that no matter how heavily I smoke outside, I don't smoke inside the house. […] From the very beginning of my smoking until I quitted it, as long as I got home, I would naturally stop smoking. […] I would go outdoors [when I am desperate for the nicotine].” (P27, male, 77)

To deal with some specific incidents, participants were expected to perform the coping strategies on demand, like helping themselves when it came to an emergency heart attack or adapting a new health behavior in daily life to get rid of risks. This was practiced occasionally, and the behavior could assist patients in maintaining their long-term health over time. P28 described that he had found the way to protect himself when he felt that they would suddenly faint:

“If I was about to fall at that moment, I would squat down, which made it safer.” (P28, male, 65)

Practicing suggested activities intermittently referred to episodic responses as well. Moreover, even though patients implemented healthier self-management regimens, some impactful events could still trigger patients' previous unadjusted self-management behaviors. These events mainly involved medication non-adherence, overeating, and smoking among male participants. Several participants described their experiences of stopping smoking and return to smoking. Most admitted that smoking due to social needs. P9 had tried to quit smoking three times, but he failed every time, and he made his mind to quit it after discharge. P5 said he had succeeded in smoking cessation. But later, he suffered the tremendous pain of losing his father. It was such a dreadful blow, so he picked cigarettes up to de-stress himself. Nonetheless, he stopped smoking after he got over this sadness:

“After seven years of quitting smoking, I restarted smoking and kept for another half a year. It has been over five months since my second smoking cessation.” (P5, male, 52)

## Discussion

### Mutual Influences

This study has shown that cardiovascular patients have immersed themselves in daily real-world contexts and experienced kinds of life events. Some repetitive self-management regimens were more like routines embedded into daily lives, and these behaviors were practiced regularly without extra reminders. Repeating the same behavior could strengthen the awareness of the initiative and reinforcement of a healthy habit. However, there have been rare qualitative studies discussing this topic. It is also worth mentioning that electronic devices have played an important role in patients' daily self-management. Patients could get access to a large amount of useful information when they get the urge to know something rather than make appointments with their physicians on selected days. This is more convenient and time-saving. Several older informants were skilled in using electronic products to assist in their self-management in this study. The report of the 41st China Statistical Report on Internet Development (27) showed that not only the young population is fond of browsing websites, but also an increasing number of the elderly are interested in looking for information purposively online. However, information selection requires further abilities, and consulting with a doctor was more reliable (28). In our study, older patients with higher educational backgrounds told more about resource utilization behaviors than those who did not obtain enough education. This implies that the development of technology is gradually adopted by a part of older people with chronic diseases as an appealing and effective cooperator even though there are some barriers (29, 30). On the other hand, there is also evidence that past educational experiences have a profound impact on the future health behaviors of self-management. It is also approved that different vocations affect the outcomes of an anticipated self-management and vice versa, which is obviously seen among the younger male informants in our study. Social expectations regard working as role responsibility within socialization, while body conditions have prevented better working activities due to time of shifts, working places, and work intensity. In line with the findings in a mixed-methods study (31), patients with heart failure continued to work due to socially based values until they were highly symptomatic.

Life changes and life-threatening experiences are both major life turning points, but the differences between them are that the former focus more on the general changing of demographic characteristics and connections with the social environment in a broader perspective, while the latter seem more personally concerned about individuals' adaptation to themselves. As referred to in the interviews, life changes mainly lead to changes in social networks, cultural atmosphere, and socioeconomic status, which are all contextual factors for self-management (32, 33). Life-threatening experiences would affect physical and psychological status. Consistent with Mariola Zapater-Fajarí and her co-workers, the more active psychological states individuals have, the better and more positively they could adapt to the self-management when they faced adverse life events (34). Emotional experiences are kind of slow and cumulative infiltration. Female participants would be more sensitive than males to this type of life experience. They suffered more from the emotional life events, so they used different coping strategies; nevertheless, they still felt unable to release their stress and had a sense of insecurity. In accord with previous studies, they might grow dependent on others and felt guilty for being sick and not correspond to gendered societal expectations (35, 36).

It could be an indication for patients that they should pay more attention to previous events that were imperceptible but have deeply affected them, which helped them understand what is “living with dying.” These life events have their repeatability, diversity, and unpredictability, which were associated with the ongoing awareness of mortality in patients living with life-threatening diseases, as the evidence showed in a previous study (37). It was the diversity and unpredictability of diverse life events that led to uncertainty of the health outcomes, and numerous results brought new life events in turn. Allender et al. (38) found that life changes in employment status, residence, physical status, relationships, and family structure could influence the engagement of physical activities that benefited the body conditions, as P1, P18, and P19 mentioned above. As a consequence, mutual influences generated various coping approaches. Therefore, kinds of feedback had been made reflecting patients' perspectives toward life events and the responses on self-management development (39).

### Coping Flexibility

This study indicated the importance of recognizing coping flexibility within the cardiovascular population. Four types of adaptive coping approaches were extracted from the qualitative data: decision-making, avoidance, consistent responses, and episodic responses. These four coping approaches do not exist on one own but combine and/or transform mutually. Similar to the results were found from the studies conducted by other researchers (8, 17), and the author Åsa Audulv identified four different types of developmental patterns, which were consistent, episodic, on-demand, and transitional (8). Patients like P12 distinguished event situations to conduct adaptation, on-demand or consistently, which is in keeping with the situation-specific theory applied in heart failure people (40).

In our study, decision-making always happened when patients received adequate stimulation, which triggered patients to undergo adjustment. As noted in previous literature, situation awareness influenced the implementation of the coping response by perceiving and understanding the events (39, 41). When participants realized the change of the environmental factors, had different emotional feelings, or underwent physical changes, they integrated much comprehensive information and resources, combined with previous experiences, prioritized the setting activities, and then made further optimal decisions accordingly. This is a complicated process involving several steps where patients weigh risks and benefits among situations, considering personal preferences and values, and sometimes the most dominant event or situation weighs the most, in support of the review conducted by Bratzke et al. (42). Meanwhile, regarding the adjustment of P12's physical activities and P28's preventative behavior, patient's ability to respond to a similar situation can be advanced, and the coping actions will be faster increasingly. This might be because patients are far familiar with this situation and excluded some inappropriate choices in previous practices, so once practices are abundant enough for patients' training, they could make good and optimal decisions under the current contexts.

Different from the existing literature, avoidance in our study was a relatively positive but autonomously refusing response, rather than negative ideational constriction, behavioral inhibition, and awareness of emotional numbness. Participants reported that this response mostly resulted from the perception of the complexity in self-management complementation, individual preference, personality, and fear of the recapture of awful past experiences. Moreover, due to some exterior factors like policy and financial conditions, patients would choose a reasonable and cost-effective method to adapt to the contemporary conditions rather than the best one, or so-called intentional avoidance. This agrees with the results that avoiding some situations reduces anxiety through the mediating effect of expectancy (43). Patients with cardiovascular disease who kept practicing consistent responses may be on account of the benefits from the behaviors, especially the visible effects, such as the remarkably decreased blood pressure and obvious weight loss. Positive feedback enhanced the motivation to insist on self-management regimens. Another condition is the fact that they realized that their performance in everyday life is under supervision by family caregivers. According to Whitehead et al., surveillance and support from families could create a context of self-management and promote adherence to daily self-care (44). Conversely, some participants would not like to be accompanied by others only if when they perceived they were in an extremely severe body condition, for some were reluctant to seek help and some were reluctant to become a burden (31).

Our examples not only showed that patients have the same approach in different situations but also revealed that patients would have multiple patterns without cross-situational consistency. In accordance with the work of Bratzke et al. and Liu et al. (42, 45), patients would prefer implementing better self-care treatments in the hospital rather than at home due to the availability of resources and less conflicting or confusing recommendations. More specifically, episodic responses are dependent on the roles that patients are playing and the conditions they get. If participants are hospitalized, they receive acute care, rely on caregivers, and have to be empowered, but when they are at home, they appear to have more autonomy and would gain a sense of control to help themselves freely. They are expected to follow every standard routine suggested by health professionals to reach a certain target in the medical institutions, while they only need to manage common symptoms without getting worse once they are outside medical systems. Besides, they could get access to more technical knowledge from the health providers rather than unknowledgeable family members unless they are experts. Also, some participants who experienced contextual disruption to work and family relationships may have a strong perception of the loss of self, while some set a clear boundary between illness-related events and normal life, which were described as the instances of amenable childlike P2 and principled P27. As found by Kralik et al. (46), living with chronic disease is an ongoing process of transition during one's life span, so participants with the chronic disease might lose or reorient themselves or shift self-identity, accounting for their different performance in coping with different life situations. Once patients have adapted to a new role of being vulnerable patients, they would realize that learning to implement preventative behaviors could reduce the horrible result caused by the bothersome chronic conditions and disease symptoms when there terrible incidents happen. As for the very irregular practices of health behaviors, it was showed in this study that no significant effects, no strict adherence, and no continuous stimuli were the reasons why participants were repeatedly adjusting their self-management strategies.

### Inequity Accumulation and Psychological Resilience

Once patients experienced too many negative life events, stress would increase and cumulate regarding the demands associated with specific situations and roles. Thus, patients might perform ineffective responses (e.g., defensive avoidance and purposive non-adherence) after inequality accumulation over time (47), especially when patients have poor communication with health professionals, continuous low autonomy, lack of information, and poor financial status. Life events are unpredictable, so the occurrence of these kinds of life events is more challenging to the resilience of patients. P17's avoidance of receiving formal treatment could be attributed to his tremendous financial burdens, poor supportive interactions as well as irritable personality, and high self-esteem, even he is receiving support from his family. In this case, patients might change their consistent responses into episodic responses, even avoid incorporating treatment, as discussed in a meta-analytic review (48). Evidence showed that anxious patients had less resilience and lower self-efficacy but present with higher self-esteem (49, 50).

We also found that those who had positive attitudes might be more willing to acquire more knowledge, learn more skills, make positive reflections, access more resources, and increase confidence to cope effectively with chronic conditions, indicating that psychological resilience mediated past life events and participants' coping approaches. Existing studies (51, 52) suggested that resilience is a remarkable protective factor for adaptability under adverse life events, and it enables cardiovascular participants to perform healthy behaviors, and promoted better outcomes, like resilient individuals P4, P5, and P8. However, P5 resumed smoking to dissolve the stress when hit by his father's passing away, probably resulting from the change in the family structure and social support (53). Not like P17, even though P8, who was of a similar age, suffered from awful life changes and got heavy financial burdens, he still demonstrated his resilience in the face of all the difficulties due to his high self-efficacy. In contrast to the other positive examples in our study, P1 was optimistic and confident to deal with his problems, yet he lacked self-efficacy to engage in self-management programs that benefited his body's health. Otherwise, P1 reported more successful experiences compared with stressful life events.

### Implications and Limitations

Everyone has unique and changing life events, and they should be carefully listened to and valued about their actual in-depth perceptions. Further study can focus on one patient's life stories in narrative inquiry to explore the development of the patient's trajectory of self-management adaptation development. It is also a good way for health professionals to develop more personal interventions precisely regarding individuals' past experiences, emotional responses, and preferences in future researches, such as recalling positive personal events of situation simulation or make self-management behaviors schedule into daily routines. Further researches could focus on building the resiliency of cardiovascular individuals by cognitive interventions who experience adverse events and empowering patients with more effective coping approaches as well as enhancement of the quality of life.

There were still several limitations that should be noted. First, the data were only analyzed by the first author but being reviewed by other research members and discussed with an expert with a different cultural background, which enhanced the study reliability. Second, qualitative data did not cover the variation in participants' self-management descriptions due to the small sample size, as well as the single recruitment site and a short period. The hospital setting might only provide a partial perspective to self-management. Additionally, individuals with cardiovascular diseases were recruited without specific illness diagnoses or trajectories. Further perceptions could be explored by targeting patients with a single cardiac disease. The dataset could be enriched by increasing recruitment sites and carrying out multiple interviews. Using the ethnographic data collecting method would generate more culture-based narratives. Patients speaking rare dialects always lack attention and understanding. Further studies could mainly focus on these groups to explore their insights about self-management. Moreover, the researchers could select participants and analyze the results regarding the disease definitions and classifications. Also, the focus group could improve the interaction and then develop more information.

## Conclusion

In conclusion, this study has provided essential insights into various life events and adaptive coping approaches for self-management reported by a group of hospitalized cardiovascular patients. Cardiovascular patients have a diversity of life events with unpredictability and uncertainty. There is a mutual influence between life events and adaptive coping approaches: the latter is developing from the former due to the diversity and unpredictability. When experiencing different life events, patients would cope flexibly regarding their situations and contexts. Due to the inequity accumulation of kinds of life events, patients will use their psychological resilience to mediate their especially emotional stress to perform adaptively through positive and negative responses. This study illuminated the importance of understanding the context, situations, and experiences about how cardiovascular patients adapt to their self-management regimens.

## Data Availability Statement

The datasets presented in this article are not readily available to protect the privacy of the participants. Requests to access the datasets should be directed to Ruolin Qiu, 11518289@zju.edu.cn.

## Ethics Statement

The studies involving human participants were reviewed and approved by The Ethics Committee, Sir Run Run Shaw Hospital, affiliated with the Zhejiang University School of Medicine. The patients/participants provided their written informed consent to participate in this study.

## Author Contributions

ZY and LT: conceptualization. RQ: formal analysis, investigation, and writing—original draft preparation. LT: resources, project administration, and funding acquisition. RQ and XW: data curation. RQ and ZY: writing—review and editing. ZY: supervision. All authors approved the submitted version.

## Funding

This work was supported by the Health Commission of Zhejiang Province under Grant 2021RC011.

## Conflict of Interest

The authors declare that the research was conducted in the absence of any commercial or financial relationships that could be construed as a potential conflict of interest.

## Publisher's Note

All claims expressed in this article are solely those of the authors and do not necessarily represent those of their affiliated organizations, or those of the publisher, the editors and the reviewers. Any product that may be evaluated in this article, or claim that may be made by its manufacturer, is not guaranteed or endorsed by the publisher.
